# Optimal Performance and Application for Seagull Optimization Algorithm Using a Hybrid Strategy

**DOI:** 10.3390/e24070973

**Published:** 2022-07-14

**Authors:** Qingyu Xia, Yuanming Ding, Ran Zhang, Huiting Zhang, Sen Li, Xingda Li

**Affiliations:** 1 Communication and Network Laboratory, Dalian University, Dalian 116622, China; xiaqingyu0315@163.com (Q.X.); nancy444@163.com (R.Z.); hwilting@163.com (H.Z.); leeson1028@163.com (S.L.); lixingdahh@163.com (X.L.); 2 School of Information Engineering, Dalian University, Dalian 116622, China

**Keywords:** seagull optimization algorithm, Sobol sequence, sigmoid function, particle swarm optimization, blind source separation

## Abstract

This paper aims to present a novel hybrid algorithm named SPSOA to address problems of low search capability and easy to fall into local optimization of seagull optimization algorithm. Firstly, the Sobol sequence in the low-discrepancy sequences is used to initialize the seagull population to enhance the population’s diversity and ergodicity. Then, inspired by the sigmoid function, a new parameter is designed to strengthen the ability of the algorithm to coordinate early exploration and late development. Finally, the particle swarm optimization learning strategy is introduced into the seagull position updating method to improve the ability of the algorithm to jump out of local optimization. Through the simulation comparison with other algorithms on 12 benchmark test functions from different angles, the experimental results show that SPSOA is superior to other algorithms in stability, convergence accuracy, and speed. In engineering applications, SPSOA is applied to blind source separation of mixed images. The experimental results show that SPSOA can successfully realize the blind source separation of noisy mixed images and achieve higher separation performance than the compared algorithms.

## 1. Introduction

Optimization has great importance and applications and is used to address complex issues to reduce computational cost, increase accuracy, and enhance performance, particularly in the field of engineering. Optimization aims to maximize efficiency, performance, and productivity through calculation under certain constraints [1]. Traditional optimization methods such as Newton and conjugate gradient methods can only deal with simple, continuously differentiable, or high-order differentiable objective functions [2,3]. With the increasing diversity and complexity of problems, the traditional optimization algorithms cannot meet the requirements of high computing speed and low error rate. Therefore, it is of great practical significance to find new optimization methods with fast calculation speed and strong convergence ability [4].

Metaheuristic algorithms have the characteristics of self-organization, mutual compatibility, parallelism, integrity, and coordination characteristics. This kind of algorithm only needs to know the objective function and the search range and can achieve the target solution regardless of whether the search range is continuously differentiable, which provides a new way to solve the optimization problem [5]. Metaheuristic algorithms belong to the stochastic optimization method and are mainly driven by the random streams (single or multiple) utilized in the stochastic search mechanism. A recent study shows that if the randomness of the random streams of interest is deliberately controlled without disturbing its expectation, then the desired effectiveness in the optimization search can be eventually gained [6]. Metaheuristic optimization algorithms are mainly divided into biological evolution, natural phenomena, and species living habits. Biological evolution methods, such as genetic algorithm (GA) [7] and differential evolution (DE) algorithm [8], are inspired by biological genetics, mutation, and evolution strategies. A natural phenomenon algorithm is a kind of algorithm based on the physical laws of nature, such as the sine cosine algorithm (SCA) [9] and biogeography-based optimization (BBO) [10]. The inspiration for the population life habit algorithm comes from the relationship between population individuals, including particle swarm optimization (PSO) [11], artificial bee colony (ABC) algorithm [12], cuckoo search (CS) algorithm [13], bat algorithm (BA) [14], and ant colony optimization (ACO) [15].

Seagull optimization algorithm (SOA) is a new metaheuristic algorithm inspired by species’ living habits proposed in 2019 [16]. SOA realizes the function of global and local search by simulating the long-distance migration behavior and foraging attack behavior of seagulls. The principle of SOA is relatively simple and easy to implement, and it has been used to address some engineering problems [17,18,19]. However, due to the low searchability of the fundamental SOA, the algorithm falls into local optimization. Therefore, improving SOA is an essential step to expanding the application scope of SOA and improving the utilization value of SOA.

In recent years, scholars have proposed many improved algorithms. Che et al. [20] introduced the reciprocity mechanism and coexistence mechanism in the symbiotic organism’s search (SOS) algorithm, which improved the development ability of the algorithm. Zhu et al. [21] applied Henon chaotic map to initialize the seagull population and combined it with differential evolution based on an adaptive formula, which improved the diversity of the seagull population. Wu et al. [22] used a chaotic tent map to initialize the population and designed a nonlinear inertia weight and random double helix formula. Experiments show that the algorithm improves the optimization accuracy and efficiency of SOA. Muthubalaji et al. [23] designed a hybrid algorithm SOSA, which uses the advantages of the owl search algorithm (OSA) to improve the global search ability of SOA. Hu et al. [24] proposed an ISOA with higher optimization accuracy, introducing non-uniform mutation and an opposite-based learning strategy. Wang et al. [25] analyzed the parameter A of SOA in detail, presented the best advantage set theory and the idea of the Yin-Yang Pair, and proposed an improved seagull fusion algorithm, YYPSOA. Ewees et al. [26] introduced Levy flight strategy and mutation operator to prevent the algorithm from falling into local optimum. Wang et al. [27] introduced the opposite-based learning strategy to initialize the population, and used the quantum optimization method to update the seagull population. The algorithm is effective in multi-objective optimization problems. The above references are some improvement methods by scholars for SOA. Although they can improve the search performance of the algorithm and reduce the premature convergence of the algorithm to a certain extent, most references only focus on the improvement of single search performance and ignore the balance between global search ability and local development ability.

This paper proposes a new SOA algorithm based on hybrid strategies named SPSOA. Firstly, the seagull population is initialized by the Sobol sequence so that the seagulls are more evenly distributed in the initial solution space. Then, through the expansion and translation of the sigmoid function, a new parameter is proposed to further enhance the algorithm’s ability to coordinate the early exploration and late development. Finally, the PSO learning strategy is introduced into the updating method of seagull attack position to enhance the ability of the algorithm to jump out of local optimization. In this paper, 12 benchmark test functions are selected to test the algorithm’s performance from different aspects. The experimental results show that the stability, convergence accuracy, and speed of SPSOA are better than other algorithms. In applying blind source separation (BSS), SPSOA can successfully separate noisy mixed images and has better separation performance than the compared algorithms.

The remainder of this paper is organized as follows: Section 2 discusses the details of the SOA. Section 3 addresses the SPSOA implementation. Section 4 verifies the effectiveness of SPSOA through experiments. Section 5 applies SPSOA to the problem of BSS of mixed images, and Section 6 concludes the paper and proposes future work.

## 2. The Basic Seagull Optimization Algorithm (SOA)

Seagulls have a natural ability to migrate and attack. Migration is a seasonal long-distance movement from one place to another. The initial position of seagulls is in different spatial areas to avoid collision during movement. In group migration, the most suitable seagull leads the migration group, and the rest of the seagulls follow this leader and update their current position in the migration process. The attack is manifested in the process of foraging, making a similar spiral action to attack the prey. SOA was used to establish a mathematical model for these two behaviors and iteratively seek the optimal solution by constantly updating the seagull positions.

### 2.1. Migration Behavior

During migration, SOA simulates how seagulls move from one location to another. At this stage, seagulls should meet three conditions.

(1) Avoid the collisions:

In order to avoid colliding with the surrounding seagulls, SOA uses variable *A* to adjust the position of each seagull.

(1) $${\vec{C}}_{S}=A\times {\vec{P}}_{S}(t)$$

where ${\vec{C}}_{S}$ represents the position where there is no collision with other seagulls, ${\vec{P}}_{S}(t)$ describes the current position of the seagull, and *t* represents the current number of iterations.

The calculation formula of variable *A* is as follows:

(2) $$A=f_{C}-f_{C}(t/T)$$

where the value $f_{C}$ is 2, *T* is the maximum number of iterations. The value of *A* decreases linearly from 2 to 0 with the increase of the number of iterations *t*.

(2) Determine the best seagull direction

After ensuring no collision between seagulls, the best direction of seagulls is calculated.

(3) $${\vec{M}}_{S}=B\times \left({\vec{P}}_{bS}(t)-{\vec{P}}_{S}(t)\right)$$

where ${\vec{M}}_{S}$ represents the direction in which the individual seagull moves to the best position, ${\vec{P}}_{bS}(t)$ represents the direction of the best seagull.

The calculation formula of variable *B* is as follows:

(4) $$B=2\times A^{2}\times rand$$

where *rand* shows a random number between 0 and 1.

(3) Move in the direction of the best seagull

After calculating the best seagull position, the seagull begins to move to this position.

(5) $${\vec{D}}_{S}=\left|{\vec{C}}_{S}+{\vec{M}}_{S}\right|$$

where ${\vec{D}}_{S}$ represents the distance between each seagull and the best position.

### 2.2. Attack Behavior

When seagulls attack the prey, they spiral in the air, constantly changing the attack angle and speed. This behavior in the *x*′, *y*′, and *z*′ planes is described as follows.

(6) $$x^{\prime }=r\times \cos(k)$$

(7) $$y^{\prime }=r\times \sin(k)$$

(8) $$z^{\prime }=r\times k$$

(9) $$r=u\times e^{kv}$$

where *r* represents the radius of each helix circle when the seagull attacks, *k* is the random number between [0,2π], *u* and *v* are the constants defining the shape of the helix, and *e* is the base of the natural logarithm.

(10) $${\vec{P}}_{S1}(t)={\vec{D}}_{S}\times x^{\prime }\times y^{\prime }\times z^{\prime }+{\vec{P}}_{bS}(t)$$

where ${\vec{P}}_{S1}(t)$ represents the attack position of seagulls.

The pseudo code of SOA is provided in Algorithm 1
**Algorithm 1: SOA**Input: Objective function *f*(x), seagull population size *N*, dimensional space *D*, maximum number of iterations *T*.1. Initialize population;
2. Set $f_{C}$ to 2;
3. Set u and v to 1;
4. **While**
*t* < *T*
5.      **for**
*i* = 1 : *N*
6.      Calculate seagull migration position ${\vec{D}}_{S}$ by Equation (5);
7.      Compute $x^{\prime }$,$y^{\prime }$,$z^{\prime }$,r using Equations (6)–(9);
8.      Calculate seagull attack position ${\vec{P}}_{S1}(t)$ by Equation (10);
9.      Update seagull optimal position ${\vec{P}}_{bS}(t)$;
10.      *t* = *t* + 1;
11.    **end for**
12. **end while**
13. Output the global optimal solution.

## 3. SPSOA Search Algorithm

### 3.1. Sobol Sequence Initialization

In a metaheuristic algorithm, the initialization population’s distribution greatly affects the algorithm’s convergence speed and accuracy [28]. When dealing with the problem of unknown distribution, the initial value of the population should be evenly distributed in the search space as much as possible to ensure high ergodicity and diversity and improve search efficiency [29]. In SOA, the random number in the search space generates the initialization population. This method has low ergodicity, uneven individual distribution, and unpredictability, which affects the algorithm’s performance to a certain extent.

To solve the above problem, some scholars use chaos search to optimize the initialization sequence [21,22,30,31,32]. Although the diversity and ergodicity of the population are improved to a certain extent, the chaotic map is greatly affected by the initial solution, and the inappropriate initial solution will lead to negative optimization of the algorithm [33].

The Sobol sequence is a low-discrepancy sequence with the advantages of short calculation cycles, fast sampling speeds, and higher efficiency in processing high-dimensional sequences [34,35]. Unlike the pseudo-random number, the low-discrepancy sequences use the deterministic low-discrepancy sequence to replace the pseudo-random sequence. By selecting a reasonable sampling direction, the points, as uniform as possible, are filled into the multi-dimensional hypercube unit. Therefore, it has higher efficiency and uniformity in dealing with probability problems. Therefore, this paper uses the Sobol sequence to map the initial population. Let the upper and lower bounds of the optimal solution be *ub* and *lb,* respectively, and the random number generated by the Sobol sequence be $S_{i}\in [0,1]$, then the mathematical model of the initialization population of the Sobol sequence is:

(11) $$x=lb+S_{i}\times (ub-lb)$$

**Figure 1:**
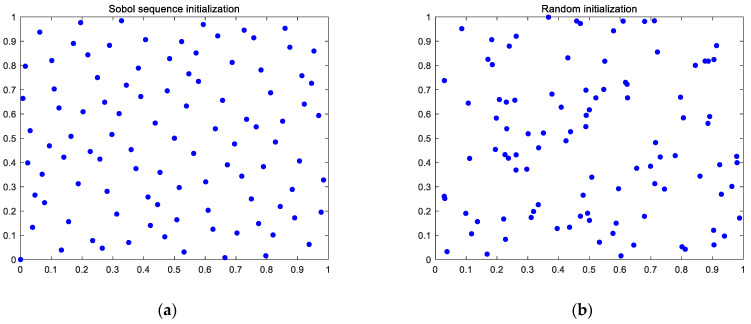
Sobol sequence initialization compared with random initialization. (**a**) Sobol sequence initialization. (**b**) Random initialization.

Let the search space dimension *D* be 2, the upper and lower bounds be 1 and 0, respectively, and the population size *N* be 100. Compare the initial population distribution of the Sobol sequence with the random initial population distribution, as shown in Figure 1.

It can be seen from Figure 1 that the spatial distribution of the Sobol sequentially initialized population is more uniform than that of the randomly initialized population, and there is no overlapping of individuals, resulting in better initial population diversity, which lays a foundation for the global search of the algorithm.

### 3.2. Improvement of Parameter A

SOA controls the frequency of parameter *A* by introducing $f_{C}$ so that the value of parameter *A* decreases linearly from 2 to 0 with the iteration to avoid the collision between individuals during the flight of seagulls and produce repeated optimization values. Parameter *A* plays a vital role in solving optimization problems and balancing algorithms. However, in practical optimization problems, the process presents a nonlinear downward trend, and the process is also highly complex. Therefore, parameter *A* of linear convergence is not fully applicable to the search process of SOA.

This paper proposes an adaptive parameter $A^{*}$ based on the sigmoid function. In this method, the value $A^{*}$ presents a nonlinear change trend in the decreasing process. In each iteration, it can avoid the position conflict between seagulls and better balance early exploration and late development. The sigmoid function can map variables between intervals [0,1], and its mathematical expression is:

(12) $$S(x)=\frac{1}{1+e^{-x}}$$

**Figure 2:**
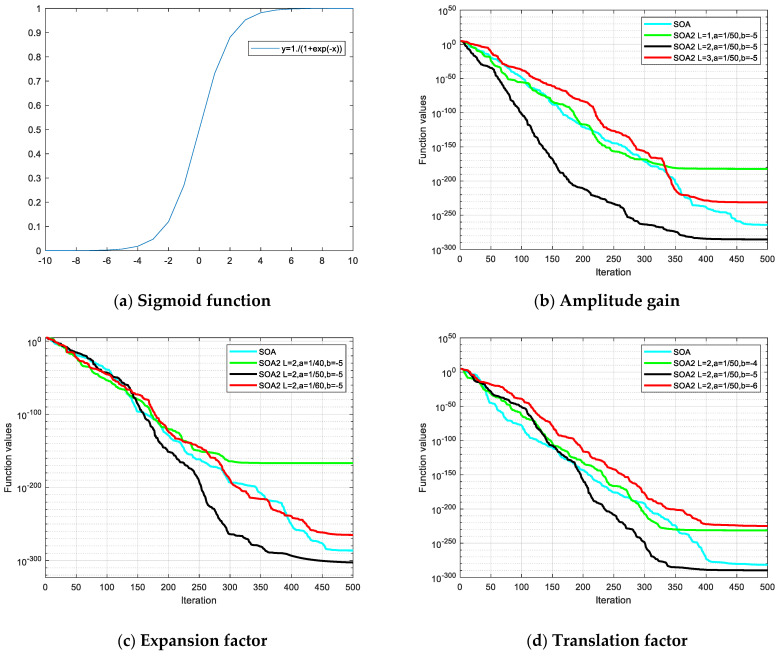
Function iteration and convergence curve. (**a**) Iteration curve of sigmoid function, (**b**) Convergence curve of Sphere for different amplitude gain values setting. (**c**) Convergence curve of Sphere for different expansion factor values setting. (**d**) Convergence curve of Sphere for different translation factor values setting.

As seen from Figure 2a, the sigmoid function is a strictly monotonically increasing, continuous, and smooth threshold function. Perform telescopic translation on Equation (12) and introduce amplitude, telescopic, and translational factors to obtain:

(13) $$S(x)=L\times \frac{1}{1+e^{ax+b}}$$

where *L* represents the amplitude gain, and *a* and *b* represent the expansion and translation factors. Figure 2b,d shows the iterative comparison between SOA2 with different parameters and basic SOA under the Sphere test function [33].

As shown in Figure 2b,d, when the maximum number of iterations *T* is 500L=2,   a=1/50,  b=−5, the search accuracy and speed of SOA2 are the highest. There is a negative optimization relative to basic BOA for some other parameters.

When L=2,   a=1/50,  b=−5, the improved parameter $A^{*}$ expression is:

(14) $$A^{*}=\frac{2}{1+e^{t/50-5}}$$

**Figure 3:**
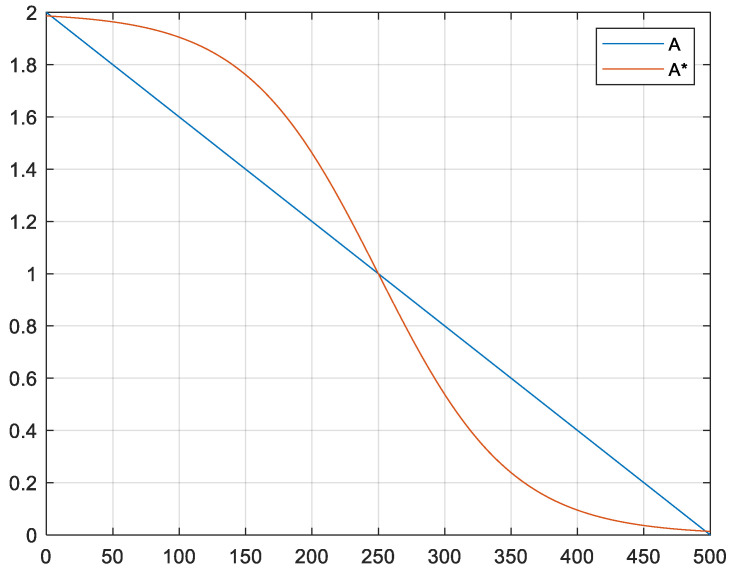
Iterative comparison curve of parameter *A* and $A^{*}$.

Figure 3 shows the iterative comparison curve of parameters A and $A^{*}$.

It can be seen from Figure 3 that parameter $A^{*}$ can make the algorithm maintain a large individual degree of freedom of the population in the early stage and enhance the global optimization ability. In the later stage, the individual degree of freedom decreases rapidly, and the local optimization ability is strengthened. Compared with parameter *A*, this paper uses the parameter $A^{*}$ to smooth the excessive migration and attack process, which can better balance early exploration and late development and make the optimization process nonlinear. Therefore, this improvement can theoretically improve the accuracy of population optimization and accelerate optimization speed.

### 3.3. Improvement of Update Function

The optimal global individual primarily guides the location update of SOA. Therefore, if the optimal global individual falls into the local optimal, the optimization is likely to stagnate. To solve this problem, this paper introduces the learning strategy in PSO, introduces the learning factor based on Equation (10), and increases the process of seagull individual learning to the optimal global position and individual historical optimal position to improve the optimization performance of the algorithm and weigh the global search and local search ability through dynamic inertia weight. The attack position update formula with learning strategy is:

(15) $${\vec{P}}_{l}(t)=({\vec{D}}_{S}\times x^{\prime }\times y^{\prime }\times z^{\prime })\times \omega +{\vec{P}}_{bS}(t)+({\vec{P}}_{bS}(t)-{\vec{P}}_{S1}(t))\times r_{1}\times c_{1}+({\vec{P}}_{GS}(t)-{\vec{P}}_{S1}(t))\times r_{2}\times c_{2}$$

(16) $$\omega =\omega_{\max}-\frac{\omega_{\max}-\omega_{\min}}{T}\times t$$

where the learning factors $c_{1}$, $c_{2}$ are set to 1.5 and $r_{1}$, $r_{2}$ are random numbers between [0,1], ω is the inertia weight, $\omega_{\max}=0.95$, $\omega_{\min}=0.35$, ${\vec{P}}_{GS}(t)$ is the direction of the best position in individual history, and ${\vec{P}}_{l}(t)$ is the attack position of seagull after learning.

By introducing the learning strategy of PSO, SPSOA updates the global optimal solution of the current population and updates the optimal historical information of each individual seagull. This can make the individual seagull jump out of the local extreme value and enter the new area of the solution space to continue to search for the optimal solution and improve the convergence accuracy and speed of the algorithm.

The pseudo code of SPSOA is provided in Algorithm 2.
**Algorithm 2: SPSOA**Input: Objective function *f*(x), seagull population size *N*, dimensional space *D*, maximum number of iterations *T*, learning factors $c_{1}$ and $c_{2}$
1. Sobol sequence initialize population;
2. Set u and v to 1;
3. **While**
*t* < *T*
4.     **for**
*i* = 1 : *N*
5.      Calculate seagull migration position ${\vec{D}}_{S}$ by Equation (5);
6.      Compute $x^{\prime }$,$y^{\prime }$,$z^{\prime }$,r using Equations (6)–(9);
7.      Calculate seagull attack position ${\vec{P}}_{S1}(t)$ by Equation (10);
8.      Compute *w* using Equation (16);
9.      Calculate learning location ${\vec{P}}_{l}(t)$ by Equation (15);
10.    Update seagull optimal position ${\vec{P}}_{bS}(t)$;
11.      *t* = *t* + 1;
12.    **end for**
13. **end while**
14. Output the global optimal solution.

### 3.4. Time Complexity Calculation

In the basic SOA, the dimension of the position-independent variable is *n*, and the population size is represented by *N*. In the initialization stage, generate a uniformly distributed random number to objectify the time to set the initial value of each parameter. Then, calculate the value of the objective function and sequence the fitness values of all individuals to obtain the contemporary optimal individual fitness value is $t_{1}$, $t_{2}$, f(n), and $t_{3}$ respectively. Then, the overall time complexity of this stage is:

(17) $$T_{1}=O(t_{1}+N\times (n\times t_{2}+f(n))+t_{3})=O(n+f(n))$$

In the collision avoidance stage of migration behavior, parameter *A* is generated from Equation (2), which changes with the number of iterations, but the value of parameter *A* is the same in the population of the same generation, so the generation time is $t_{4}$. According to Equation (1), the time for updating the position of the individual seagull in each dimension is $t_{5}$, and the calculation time of the new seagull fitness value is f(n), then the time complexity of this stage is:

(18) $$T_{2}=O(N\times (t_{4}+n\times t_{5}+f(n)))=O(n+f(n))$$

In calculating the best seagull direction of migration behavior, parameter *B* is generated from Equation (4), and the value of parameter *B* in the same generation population is the same, and its generation time is $t_{6}$. According to Equation (3), the time for updating the position of the seagull individual in each dimension is $t_{7}$, and the calculation time of the new seagull fitness value is f(n), then the time complexity of this stage is:

(19) $$T_{3}=O(N\times (t_{6}+n\times t_{7}+f(n)))=O(n+f(n))$$

In the stage of moving towards the best seagull direction of migration behavior, the time of generating each one-dimensional element in the new individual according to Equation (5) is $t_{8}$, and the calculation time of the new fitness value is f(n), then the time complexity of this stage is:

(20) $$T_{4}=O(N\times (n\times t_{8}+f(n)))=O(n+f(n))$$

In the stage of seagull attack behavior, the time required to calculate $x^{\prime }$,$y^{\prime }$,$z^{\prime }$, and r according to Equations (6)–(9) is $t_{9}$, the time of updating according to Equation (10) is $t_{10}$, and the time of calculating the new fitness is f(n), then the time complexity of this stage is:

(21) $$T_{5}=O(N\times (t_{9}+n\times t_{10}+f(n)))=O(n+f(n))$$

In the phase of updating the optimal solution, assuming that the replacement time of each fitness value compared with the current optimal solution is $t_{11}$, the time complexity of this phase is:

(22) $$T_{6}=O(N\times t_{11})=O(t_{11})$$

To sum up, the total time complexity of SOA is:

(23) $$T(n)=T_{1}+T\times (T_{2}+T_{3}+T_{4}+T_{5}+T_{6})=O(n+f(n))$$

where *T* is the maximum number of iterations.

In SPSOA, the dimensions of population size and location independent variables are entirely consistent with the basic SOA. In the initialization stage, the time for parameter setting, solving, and sorting the fitness value of the objective function and obtaining the contemporary optimal individual fitness value is also the same as that of SOA. The time for generating the random number of Sobol sequence is $t_{12}$, so the time complexity of this stage is:

(24) $$T_{1}^{*}=O(t_{1}+N\times (n\times t_{12}+f(n))+t_{3})=O(n+f(n))$$

An adaptive parameter based on sigmoid function is introduced in the migration behavior collision avoidance stage of SPSOA. Its generation time is $t_{13}$, and the generation time of new seagull individuals is $t_{14}$. The parameter also changes with the number of iterations, and the value in the same generation population is the same. The remaining time of this stage and the time of calculating the best seagull direction, moving to the best seagull direction, and updating the optimal solution are the same as those of SOA. Therefore, the time complexity of the SPSOA migration stage is:

(25) $$T_{2}^{*}=O(N\times (t_{13}+n\times t_{14}+f(n)))+T\times (T_{3}+T_{4}+T_{6})=O(n+f(n))$$

In the stage of SPSOA attack behavior, the learning strategy of PSO is introduced. The location update time is $t_{15}$, and the remaining time is the same as SOA. Therefore, the time complexity of the SPSOA attack phase is:

(26) $$T_{3}^{*}=O(N\times (t_{9}+n\times (t_{10}+t_{15})))=O(n+f(n))$$

To sum up, the total time complexity of SPSOA is:

(27) $$T(n)=T\times (T_{1}^{*}+T_{3}^{*})+T_{2}^{*}=O(n+f(n))$$

According to the analysis in this section, compared with the basic SOA, SPSOA does not add additional time complexity, the two are exactly the same, and the execution efficiency does not decrease.

## 4. Simulation and Result Analysis

In this section, to verify the performance of SPSOA more comprehensively, 12 benchmark test functions are used for experiments. The experimentation is divided into two parts: the first part compares the three improvement strategies proposed in this paper with SPSOA and basic SOA, respectively, proving that these improvement strategies are effective. The second one compares SPSOA with other metaheuristic algorithms to verify that the search performance of SPSOA is better than the compared algorithm. To ensure the fairness of the experimental results, each algorithm was performed separately 30 times to minimize the error, and all tests were conducted on a laptop equipped with an Intel (R) Core (TM) i7-6500 CPU at 2.50 GHz and 8 GB of RAM. The population size *N* of all experiments is 30, and the maximum number of iterations *T* is 500.

The detailed characteristics of each test function are listed in Table 1. In Table 1, Dim denotes the function dimension, Scope represents the value range of *x*, and *f*_min_ indicates the ideal value of each function.

### 4.1. Effectiveness Analysis of Improvement Strategy

The SPSOA proposed in this paper is a hybrid algorithm based on SOA using three strategies. However, it is not known whether any strategy will work, so it needs to be verified. In this part, SOA1 (introduce Sobol sequence initialization), SOA2 (design new parameter $A^{*}$), and SOA3 (introduce the learning strategy of PSO) are compared with SPSOA and basic SOA. Table 2 shows the optimal fitness value (BEST), the worst fitness value (WORST), the average fitness value (MEAN), and the standard deviation (STD) of 30 experiments of each algorithm under the 12 test functions in Table 1. In Table 2, *Dim* denotes the function dimension, *Scope* represents the value range of *x*, *f*_min_ indicates the ideal value of each function, and the best test results of all algorithms are in bold.

According to Table 2, the indexes of SOA1, SOA2, and SOA3 proposed in this paper improved to varying degrees compared with the basic SOA. In the three test functions, *F*_1_, *F*_7,_ and *F*_8_, all the algorithms can find the theoretical optimal value. However, from the MEAN and WORST of *F*_1_, it can be seen that SOA1, SOA2, and SOA3 have better stability than the basic SOA. In *F*_4_, all algorithms are prone to falling into local optimum, but the BEST, WORST, and MEAN of SPSOA are better than other algorithms, especially BEST, which is significantly improved. However, the STD of SPSOA is higher than that of other algorithms. This is because the characteristics of *F*_4_ lead to low search accuracy in most cases, so the experimental results are within a reasonable range. Based on the data shown in Table 2, the three improvement strategies proposed in this paper are effective and have a stable improvement in the convergence speed, convergence accuracy, and jumping out of the local optimum of the algorithm. In other test functions, the improved SPSOA with a mixed strategy is better than the enhanced algorithm with a single strategy in solving the four evaluation indexes, which shows that the optimization ability and stability of the algorithm are improved to a greater extent under the joint influence of different strategies.

**Figure 4:**
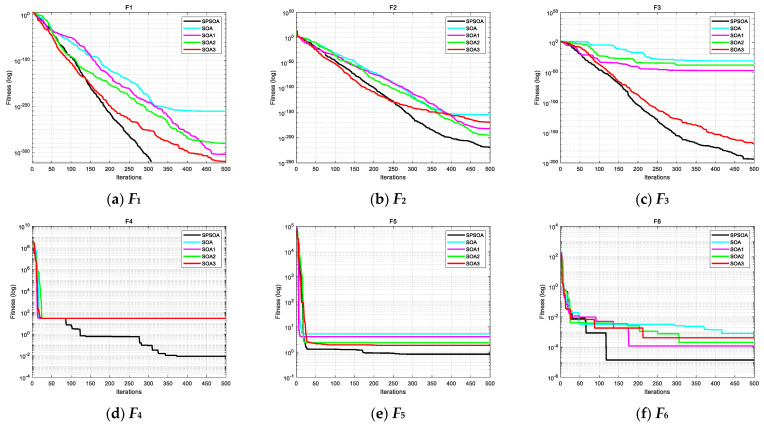

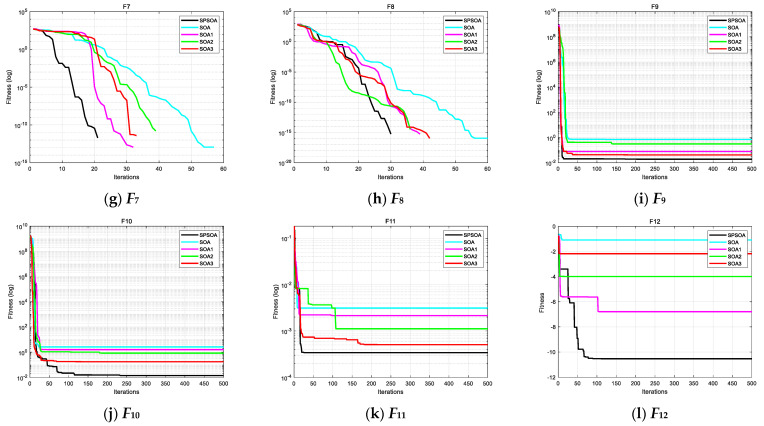
Convergence curves of the SOA and its improved algorithms for the 12 test functions.

Since each algorithm in some test functions has a strong optimization ability and cannot reflect the role of each strategy, further explanation and analysis are required. As shown in Figure 4 with the two test functions, *F*_7_ and *F*_8_, although SOA can also converge to the theoretical optimum, it is not as good as other algorithms in terms of search speed. SOA1, SOA2, and SOA3 improved by three single strategies are better than the basic SOA in convergence speed and optimization accuracy but inferior to the SPSOA improved by mixed strategies. It shows that each strategy fully plays its role and is effective. SOA1 introduces the Sobol sequence to ensure the initial population’s diversity and evenly distribute the search space. SOA2 designs a new parameter based on the sigmoid function, which is more suitable for the nonlinear iterative process of the algorithm and coordinates the global search and local search of the algorithm. After SOA3 introduces PSO learning strategy, the ability to jump out of local optimization and convergence speed of the algorithm is enhanced, which further verifies the effectiveness of the three hybrid strategies proposed in this paper.

### 4.2. Comparative Analysis of Algorithm Performance

To verify the superiority and feasibility of SPSOA, this part adopts six optimization algorithms: MSOA [36], BSOA [37], PSO [11], GWO [38], WSO [39], and BOA [40], and makes a comprehensive comparison with SPSOA under the 12 test functions in Table 1. The parameters of other algorithms are consistent with those in the corresponding references. The experiment in Table 3 adds the algorithm running time (TIME) based on Table 2, in which the time unit is second. The best test results of all algorithms have been highlighted in Table 3. In Section 4.1, it has been proven that SPSOA has better performance than the basic SOA, so the SOA is not added for comparison in the following comparative experiment.

It can be seen from the test results in Table 3 that SPSOA is optimal in the three indexes of BEST, WORST, and MEAN of all test functions. It shows that the global search ability and local development ability of SPSOA are better than the compared algorithms. In *F*_1_, BSOA can find the theoretical optimal value, but it is inferior to SPSOA in WORST and MEAN. In *F*_7_ and *F*_8_, the performance indexes of MSOA and BSOA are as excellent as SPSOA. Although in *F*_4_, SPSOA is worse than GWO and WOA in STD, it performs better in other indexes, especially in BEST. This is because the function makes the algorithm fall into local optimization, and the excellent global search ability of SPSOA improves the probability of jumping out of local optimization in the iterative process. As for the calculation time in Table 3, SPSOA has the smallest execution time in all test functions, which shows that the convergence speed of SPSOA is better than the compared algorithms and can be adopted to a variety of real-time environments.

**Figure 5:**
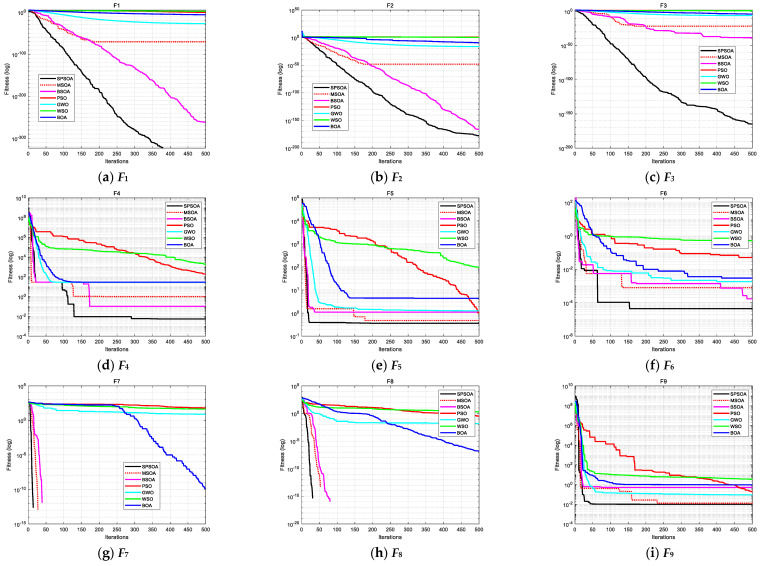

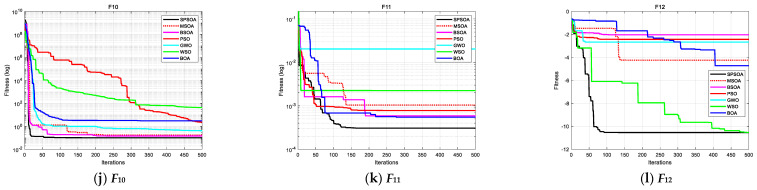
Convergence curves of 7 intelligence algorithms for the 12 test functions.

To more intuitively display the convergence speed and optimization accuracy of the algorithm and show the ability of the algorithm to jump out of the local optimization, Figure 5 gives the convergence curves of 12 test functions according to the number of iterations and fitness value. In *F*_7_ and *F*_8_, MSOA and BSOA can also search the optimal solution, but the number of iterations is more significant than that of SPSOA. The search speed of PSO is slow in the early iteration of the algorithm. The overall convergence performance of GWO is mediocre. The search performance of WSO and BOA increases as the function complexity increases.

To further evaluate the performance of the algorithm, under the significance level of α=5%, the Wilcoxon signed rank sum test was performed on the best results of SPSOA and 6 other algorithms under 30 independent operations [41]. We used the *p* value of the test result to compare whether there were differences between the two algorithms. When p<0.05, it indicates that there are significant differences between the two algorithms; when p>0.05, it shows that the optimization performance of the two algorithms is the same. The result analysis is shown in Table 4. The symbol “+”, “=“, “−” indicates that the performance of SPSOA is better than, equivalent to, and worse than the compared algorithms, respectively, and NaN indicates that the algorithm result is close and cannot be judged for significance.

By analyzing the results in Table 4, it can be found that in *F*_1_, *F*_7_, and *F*_8_, SPSOA has the same performance as MSOA and BSOA, both of which can find the optimal solution, but SPSOA is better in convergence speed and stability. The other *p* values are basically less than 0.05, indicating that the performance of the algorithm is statistically significant, which indicates that SPSOA has better advantages than the compared algorithms.

## 5. Application of SPSOA in Blind Source Separation

### 5.1. Basic Theory of Blind Source Separation

Blind source separation (BSS), sometimes referred to as blind signal processing, is capable of recovering the source signal from the observed signal in the absence of critical information such as source and channel [42]. Among them, blind image separation is the process of estimating or separating the original source image from the fuzzy image features. It mainly eliminates or minimizes the degradation of the image caused by interference and noise through the prior knowledge of image degradation [43].

The linear mixed BSS model is described below:

(28) X(t)=AS(t)+N(t)

where *t* is the sampling moment, *A* is a mixed matrix of order m×n (m≥n), *X*(*t*) is a vector of the m-dimensional observed signals,$X(t)=[X_{1}(t),X_{2}(t),\ldots ,X_{m}(t)]$, *S*(*t*) is a vector of the *n*-dimensional source signals, $S(t)=[S_{1}(t),S_{2}(t),\ldots ,S_{n}(t)]$, and *N*(*t*) is a vector of the *m*-dimensional noise signals. BSS represents the cases in which an optimization algorithm determines the separation matrix, *W*, when only the observed signals, *X*(*t*), are known.

The separated signals, *Y*(*t*), are obtained using Equation (2).

(29) Y(t)=WX(t)

where $Y(t)=[Y_{1}(t),Y_{2}(t),\ldots ,Y_{n}(t)]$.

**Figure 6:**
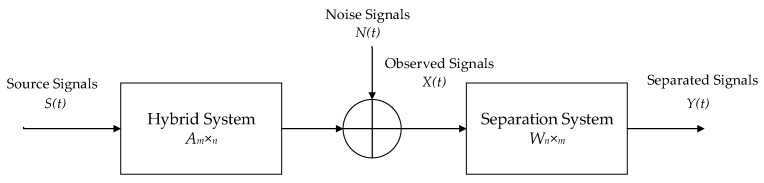
Linear mixed blind source separation model.

Figure 6 shows the linear mixed BSS model.

Independent component analysis (ICA) is an important BSS method [44]. ICA means that under the condition that the source signals are independent of each other, the appropriate signal independence criterion is used to establish the objective function. The optimal separation matrix is obtained through iterative optimization to maximize the independence of the separated signals.

The commonly used independence criterion of signals includes mutual information, kurtosis, and negative entropy. Kurtosis is calculated using Equation (30) as follows:

(30) $$K(y_{i})=kurt(y_{i})=E\{y_{i}^{4}\}-3(E{\left\{y_{i}^{2}\right\}}^{2})$$

where *y_i_* is a Gaussian random variable.

The sum of absolute values of kurtosis is used as a criterion of signal independence in this paper, and the objective function is specified as follows:

(31) $$fit_{i}=\frac{1}{\sum_{i=1}^{n}\left|K(y_{i})\right|+\varepsilon }$$

where ε is an extremely small amount that prevents division by zero. According to the information theory, for a Gaussian random vector *y_i_*, when $E[yy^{T}]=I$ the larger the kurtosis of the signals, the greater their independence. The SPSOA, as mentioned above, will optimize the separation matrix W, maximize the kurtosis, and finally complete the separation of the observed signals.

Before the iterative optimization of the objective function, it is also necessary to preprocess the observed signal, such as centralization and whitening, which can reduce the algorithm’s complexity and make a single observation signal statistically independent.

**Figure 7:**
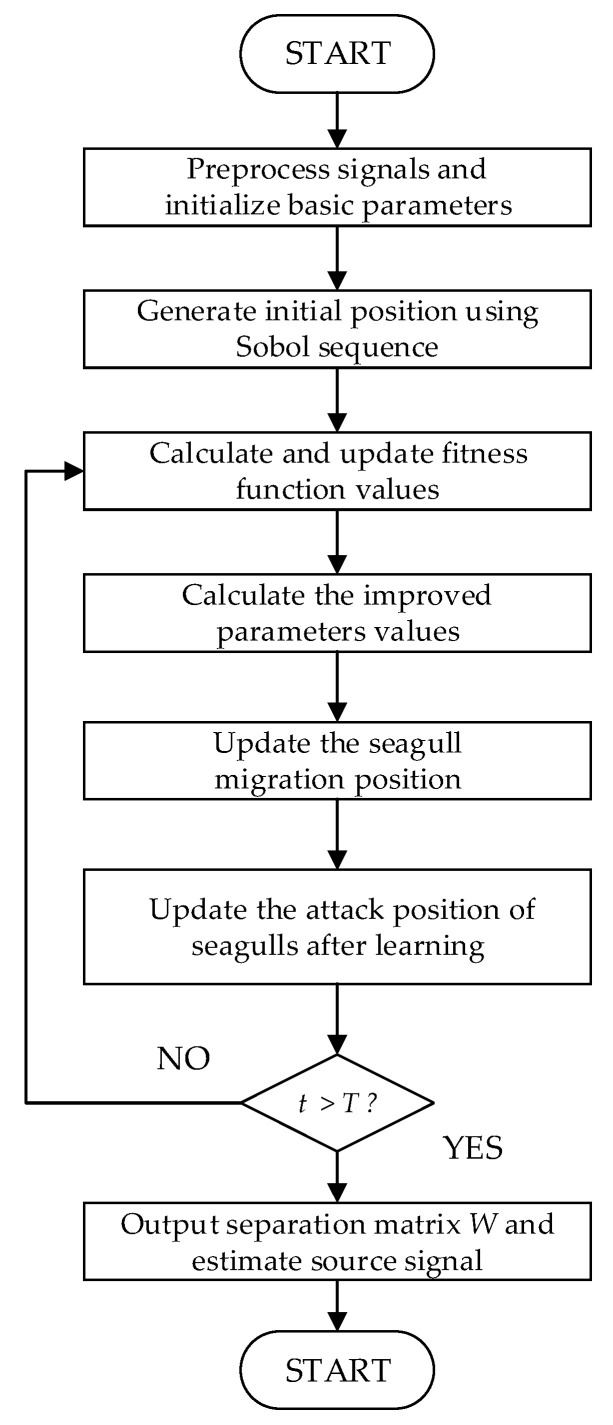
The flow chart of SPSOA-ICA.

Figure 7 shows the flow chart of SPSOA-ICA.

### 5.2. Image Signal Separation

**Figure 8:**
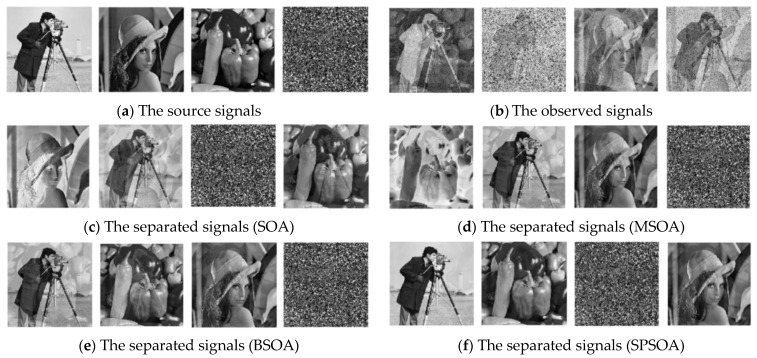
Effect drawing of image signal separation. (**a**) The image of source signals; (**b**) the image of observed signals; (**c**) the image of SOA-separated signals; (**d**) the image of MSOA-separated signals; (**e**) the image of BSOA-separated signals; (**f**) the image of SPSOA-separated signals.

Three gray-scale images and one random noise image were used as source signals and combined to produce the observed signals. To acquire the separated signals, SPSOA, SOA, BSOA, and MSOA were used to separate the observed signals blindly. The simulation diagram is depicted in Figure 8.

In order to quantitatively analyze and compare the separation performance of the four algorithms, Table 5 compares the similarity coefficient, performance index (PI) of separated signals, and the SSIM of an output image. The data results in Table 5 are the average values under multiple experiments.

(32) $$\rho_{ij}=\frac{\left|\sum_{i=1}^{N}s_{i}(t)y_{j}(t)\right|}{\sqrt{\sum_{i=1}^{N}s_{i}^{2}(t)\sum_{t=1}^{N}y_{j}^{2}(t)}}$$

(33) $$PI=\frac{1}{N(N-1)}\sum_{i=1}^{N}\{(\sum_{i=1}^{N}\frac{\left|G_{ij}\right|}{\max_{i}|G_{il}|}-1)+(\sum_{j=1}^{N}(\frac{\left|G_{ij}\right|}{\max_{j}|G_{il}|}-1)\}$$

(34) $$\mathrm{SSIM}=\frac{\left(2\mu_{\hat{x}}\mu_{x}+C_{1}\right)\left(2\sigma_{\hat{x}x}+C_{2}\right)}{\left(\mu_{\hat{x}}^{2}+\mu_{x}^{2}+C_{1}\right)\left(\sigma_{\hat{x}}^{2}+\sigma_{x}^{2}+C_{2}\right)}$$

In Equation (32), $\rho_{ij}$ is a similarity index used to compare the source signal with the separated signal. The greater the $\rho_{ij}$, more effective the separation. In this section, $\rho_{ij}$ is a 4×4 matrix. The maximum value of each channel is taken as the experimental data, and *N* is set to 4. In Equation (33) G=WA, the closer the PI is to 0, the more similar the separated signal is to the source signal. In Equation (34), $C_{1}$ and $C_{2}$ are constant, $\sigma_{\hat{x}x}$ represents the covariance of the image, $\mu_{\hat{x}}^{}$ represents the mean value of the two images, respectively, and represents the variance of two images, respectively. SSIM is in [0,1], which is a value closer to one indicating better structure preservation.

As shown in Table 5, SPSOA produces not only the highest similarity coefficient and SSIM but also the smallest PI of the separated signals, allowing for a more accurate restoration of the source signals.

It can be seen from Figure 8 that the separated signal obtained by SPSOA proposed in this paper can restore the source signal better, and its image features are similar to the source image, which can reduce the degradation of the image caused by noise. However, the separated signals obtained by other algorithms have different degrees of distortion. In addition, the sequence of the separated signals is inconsistent with the source signals, which is caused by the ambiguity of the BSS. However, in most scientific research and production practices, the ambiguity of BSS will not have a significant impact on the results.

## 6. Conclusions and Future Work

This paper proposes a hybrid strategy to improve SPSOA, which is a great improvement on the basic SOA. The algorithm uses the Sobol sequence to initialize the population, which improves the diversity of the initial population and lays a foundation for the global search of the algorithm. Using the sigmoid function to improve parameters can better adapt to the nonlinear optimization process of the algorithm and enhance the ability of the algorithm to coordinate the early exploration and later development. The learning strategy of PSO is introduced to increase the process of seagull learning from the optimal global position and individual historical optimal position, and improve the ability of the algorithm to jump out of the optimal local position. Moreover, compared with the basic SOA, SPSOA does not increase the time complexity of the algorithm. According to the simulation results, we draw the following conclusions:

(1) When optimizing 12 benchmark functions, SPSOA outperforms the other 6 algorithms. The three improvement methods proposed in this study increased the performance of SOA to varying degrees in the algorithm ablation experiment. All of this demonstrates that SPSOA has a high level of search performance and strong robustness.
(2) In BSS, SPSOA can successfully separate noisy mixed images. In addition, the algorithm is superior to the compared algorithms in the SSIM of output images, similarity coefficient, and PI of separated signals. SPSOA has a broad application prospect in modern signal processing.

In the future, the proposed algorithm can be used to solve more engineering problems, such as path planning, data compression, and resource allocation. In addition, the capability of SPSOA in solving multi-objective optimization problems needs further research.

## Figures and Tables

**Table 1:** Basic information of benchmark test functions.

Function	Dim	Scope	*f*_min_
$F_{1}(x)=\sum_{i=1}^{n}x_{i}^{2}$	30	[−100,100]	0
$F_{2}(x)=\sum_{i=1}^{n}\left|x_{i}\right|+\prod_{i=1}^{n}{|x}_{i}|$	30	[−10,10]	0
$F_{3}(x)=\max_{i}\left\{\;\left|x_{i}\right|,\;1\leq i\leq n\right\}$	30	[−100,100]	0
$F_{4}(x)=\sum_{i=1}^{n}\left[100{\left(x_{i+1}-x_{i}^{2}\right)}^{2}+{\left(x_{i}-1\right)}^{2}\right]$	30	[−30,0]	0
$F_{5}(x)=\sum_{i=1}^{n}{\left(\left[x_{i}+0.5\right]\right)}^{2}$	30	[−100,100]	0
$F_{6}(x)=\sum_{i=1}^{n}ix_{i}^{4}+\mathrm{random}[0,1)$	30	[−1.28,1.28]	0
$F_{7}(x)=\sum_{i=1}^{n}\left[x_{i}^{2}-10\cos\left(2\pi x_{i}+10\right)\right]$	30	[−5.12,5.12]	0
$F_{8}(x)=\frac{1}{4000}\sum_{i=1}^{n}x_{i}^{2}-\prod_{i=1}^{n}\cos\left(\frac{x_{i}}{\sqrt{i}}\right)+1$	30	[−600,600]	0
$\begin{array}{l} F_{9}(x)=\frac{\pi }{n}\left\{10\sin\left(\pi y_{1}\right)+{\sum_{i=1}^{n}\left(y_{i}-1\right)}^{2}\left[1+10\sin^{2}\left(\pi y_{i+1}\right)\right]+{\left(y_{n}-1\right)}^{2}\right\}+\sum_{i=1}^{n}u\left(x_{i},10,100,4\right),\;\\ \;\;\;\;\;\;\;\;\;\;\;\;\;\;\;\;\;\;\;\;\;\;\;\;\;\;\;\;\;\;\;\;\;\;\;\;\;\;\;\;\;\;\;\;\;\;\;\;\;\;\;\;\;\;\;\;\;\;y_{i}=1+\frac{x_{i}+1}{4}u\left(x_{i},a,k,m\right)=\left\{\begin{array}{l} k{\left(x_{i}-a\right)}^{m}\;\;\;\;\;\;\;x_{i}>a\\ 0\;\;\;\;\;\;\;\;\;\;\;\;\;\;\;\;\;\;-a<x_{i}<a\\ k{\left(-x_{i}-a\right)}^{m}x_{i}<-a \end{array}\right. \end{array}$	30	[−50,50]	0
$F_{10}(x)=0.1\left\{\sin^{2}\left(3\pi x_{i}\right)+\sum_{i=1}^{n}{\left(x_{i}-1\right)}^{2}\left[1+\sin^{2}\left(3\pi x_{i}+1\right)\right]+{\left(x_{n}-1\right)}^{2}\left[1+\sin^{2}\left(2\pi x_{n}\right)\right]\right\}+\sum_{i=1}^{n}u\left(x_{i},5,100,4\right)$	30	[−50,50]	0
$F_{11}(x)=\sum_{i=1}^{11}\left[ai-\frac{x1\left(bi^{2}+bix2\right)}{bi^{2}+bix3+x4}\right]$	4	[−5,5]	0.00030
$F_{12}(x)=-\sum_{i=1}^{10}{\left[\left(X-a_{i}\right){\left(X-a_{i}\right)}^{T}+c_{i}\right]}^{-1}$	4	[0,10]	−10.5363

**Table 2:** Comparative analysis of SOA and its improved algorithms.

Function	Index	SPSOA	SOA	SOA1	SOA2	SOA3
F1	BEST	**0**	0	0	0	0
	WORST	**1.94 × 10^−245^**	1.35 × 10^−192^	3.19 × 10^−237^	5.12 × 10^−217^	4.40 × 10^−241^
	MEAN	**2.84 × 10^−247^**	3.84 × 10^−194^	1.01 × 10^−239^	1.38 × 10^−219^	4.78 × 10^−243^
	STD	**0**	0	0	0	0
F2	BEST	**8.24 × 10^−259^**	2.42 × 10^−184^	6.89 × 10^−221^	4.00 × 10^−210^	7.87 × 10^−240^
	WORST	**2.77 × 10^−172^**	3.86 × 10^−133^	6.75 × 10^−168^	8.78 × 10^−137^	1.31 × 10^−152^
	MEAN	**4.24 × 10^−173^**	3.33 × 10^−135^	1.33 × 10^−169^	6.08 × 10^−139^	1.01 × 10^−154^
	STD	**0**	3.58 × 10^−134^	0	5.88 × 10^−138^	7.08 × 10^−153^
F3	BEST	**6.90 × 10^−248^**	1.72 × 10^−59^	1.61 × 10^−62^	2.09 × 10^−60^	4.50 × 10^−234^
	WORST	**2.81 × 10^−118^**	2.86 × 10^−8^	1.40 × 10^−9^	6.76 × 10^−11^	1.00 × 10^−117^
	MEAN	**1.39 × 10^−119^**	9.58 × 10^−10^	4.78 × 10^−11^	2.28 × 10^−12^	3.35 × 10^−119^
	STD	**1.62 × 10^−118^**	5.23 × 10^−9^	2.56 × 10^−10^	1.23 × 10^−11^	2.83 × 10^−118^
F4	BEST	**6.30 × 10^−4^**	28.7313	28.7208	28.7117	28.7098
	WORST	**28.8408**	28.9163	28.9036	28.9134	28.8763
	MEAN	**20.2261**	28.8028	28.7897	28.7927	28.7825
	STD	**10.2558**	0.0395	0.0364	0.0388	**0.0352**
F5	BEST	**0.0115**	0.8335	0.5901	0.3125	0.0218
	WORST	**3.0266**	5.0777	4.6241	4.3920	4.0115
	MEAN	**1.3783**	2.5841	2.5029	2.4464	1.4107
	STD	**0.9106**	1.4569	0.9351	1.3293	1.3016
F6	BEST	**2.89 × 10^−7^**	9.43 × 10^−5^	5.92 × 10^−6^	3.78 × 10^−5^	1.86 × 10^−6^
	WORST	**4.42 × 10^−4^**	0.0031	8.08 × 10^−4^	0.0018	5.32 × 10^−4^
	MEAN	**1.88 × 10^−4^**	7.57 × 10^−4^	2.23 × 10^−4^	6.01 × 10^−4^	2.67 × 10^−4^
	STD	**1.12 × 10^−4^**	7.37 × 10^−4^	2.04 × 10^−4^	4.65 × 10^−4^	1.47 × 10^−4^
F7	BEST	**0**	0	0	0	0
	WORST	**0**	0	0	0	0
	MEAN	**0**	0	0	0	0
	STD	**0**	0	0	0	0
F8	BEST	**0**	0	0	0	0
	WORST	**0**	0	0	0	0
	MEAN	**0**	0	0	0	0
	STD	**0**	0	0	0	0
F9	BEST	**3.85 × 10^−4^**	0.0201	0.0021	0.0166	7.44 × 10^−4^
	WORST	**0.1239**	1.3573	0.7825	0.7481	0.5928
	MEAN	**0.0425**	0.3687	0.3507	0.2640	0.0964
	STD	**0.0364**	0.2880	0.2364	0.2009	0.1321
F10	BEST	**1.21 × 10^−5^**	0.1531	0.0869	0.1195	8.17 × 10^−4^
	WORST	**1.5300**	2.5146	2.2179	2.4815	1.6547
	MEAN	**0.3992**	1.2135	0.8381	1.1573	0.5003
	STD	**0.4592**	0.6591	0.4602	0.6372	0.4570
F11	BEST	**3.09 × 10^−4^**	3.73 × 10^−4^	3.39 × 10^−4^	3.31 × 10^−4^	3.13 × 10^−4^
	WORST	**2.22 × 10^−3^**	0.0124	0.0067	0.0117	0.0032
	MEAN	**8.46 × 10^−4^**	0.0033	0.0025	0.0022	0.0012
	STD	**7.79 × 10^−4^**	0.0032	0.0024	0.0022	8.50 × 10^−4^
F12	BEST	**−10.5363**	−4.5193	−4.5585	−4.8779	−5.7062
	WORST	**−3.5611**	−0.1950	−1.3644	−0.8549	−1.1030
	MEAN	**−6.9625**	−1.7858	−2.9798	−3.0082	−3.8766
	STD	**1.0408**	3.0935	1.3543	2.2399	2.4978

**Table 3:** Comparative analysis of SPSOA and other optimization algorithms.

Function	Index	SPSOA	MSOA	BSOA	PSO	GWO	WSO	WOA
F1	BEST	**0**	1.09 × 10^−130^	0	0.0908	2.69 × 10^−29^	83.5621	1.78 × 10^−7^
	WORST	**1.94 × 10^−245^**	1.10 × 10^−59^	2.73 × 10^−221^	2.4206	2.08 × 10^−26^	606.1327	5.87 × 10^−7^
	MEAN	**2.84 ×10^−247^**	5.36 × 10^−61^	9.11 × 10^−223^	0.5532	1.65 × 10^−27^	257.9395	3.28 × 10^−7^
	STD	**0**	2.18 × 10^−60^	0	0.59168	3.87 × 10^−27^	124.2097	9.28 × 10^−6^
	TIME	**0.1084**	0.1241	0.1443	1.014	0.2210	0.2883	0.1894
F2	BEST	**8.24 × 10^−259^**	1.36 × 10^−77^	1.86 × 10^−205^	0.0358	2.65 × 10^−17^	1.9215	9.28 × 10^−13^
	WORST	**2.77 × 10^−172^**	2.52 × 10^−29^	7.81 × 10^−155^	20.0785	3.53 × 10^−16^	8.1539	1.32 × 10^−8^
	MEAN	**4.24 × 10^−173^**	8.42 × 10^−31^	2.60 × 10^−156^	1.7606	1.32 × 10^−16^	5.0475	6.71 × 10^−10^
	STD	**0**	4.61 × 10^−30^	1.42 × 10^−155^	4.6023	8.26 × 10^−17^	1.3673	2.40 × 10^−9^
	TIME	**0.1256**	0.1461	0.1605	0.8429	0.1401	0.1814	0.1354
F3	BEST	**6.90 × 10^−248^**	1.47 × 10^−43^	9.33 × 10^−214^	6.0333	5.62 × 10^−8^	10.48	5.39 × 10^−5^
	WORST	**2.81 × 10^−118^**	2.94 × 10^−12^	1.84 × 10^−31^	11.8971	1.88 × 10^−6^	16.46	1.05 × 10^−4^
	MEAN	**1.39 × 10^−119^**	1.06 × 10^−13^	6.13 × 10^−33^	8.6624	5.21 × 10^−7^	13.80	8.20 × 10^−5^
	STD	**1.62 × 10^−118^**	5.37 × 10^−13^	3.36 × 10^−32^	1.4717	4.33 × 10^−7^	1.72	1.37 × 10^−5^
	TIME	**0.1182**	0.1420	0.1422	0.8460	0.1402	0.1867	0.1278
F4	BEST	**6.30 × 10^−4^**	2.87 × 10^−2^	0.0829	75.3648	26.1669	2992.658	28.8767
	WORST	**28.8408**	28.8536	28.8475	90237.8870	28.7378	90507.1557	28.9532
	MEAN	**20.2261**	24.9397	26.6308	27185.0674	27.3274	19976.6055	28.9085
	STD	**10.2558**	12.388	12.1404	41931.1362	0.6798	19264.1293	**0.0182**
	TIME	**0.1503**	0.1546	0.1644	0.9514	0.1891	0.2117	0.1834
F5	BEST	**0.0115**	0.0169	0.0641	0.0570	0.1197	138.9501	4.8619
	WORST	**3.0266**	3.2671	4.3176	3.2801	3.5117	695.5827	6.3001
	MEAN	**1.3783**	1.7443	2.1365	1.6012	1.7379	313.6182	5.746
	STD	**0.9106**	1.5751	1.3466	1.4739	1.3640	141.1462	2.3374
	TIME	**0.1163**	0.1557	0.1434	0.8476	0.1574	0.1946	0.1453
F6	BEST	**2.89 × 10^−7^**	5.75 × 10^−5^	6.52 × 10^−6^	0.0289	7.29 × 10^−4^	0.0541	6.58 × 10^−4^
	WORST	**4.42 × 10^−4^**	0.0041	7.61 × 10^−4^	0.0935	0.0038	0.2165	0.0039
	MEAN	**1.88 × 10^−4^**	0.0012	2.84 × 10^−4^	0.0586	0.0020	0.1265	0.0018
	STD	**1.12 × 10^−4^**	9.67 × 10^−4^	2.06 × 10^−4^	0.0192	7.69 × 10^−4^	0.0500	8.42 × 10^−4^
	TIME	**0.1929**	0.2252	0.2279	0.9431	0.2201	0.2623	0.2884
F7	BEST	**0**	0	0	24.5566	5.68 × 10^−14^	29.2575	1.70 × 10^−13^
	WORST	**0**	0	0	97.0660	11.5549	83.9381	2.34 × 10^−8^
	MEAN	**0**	0	0	56.1656	2.2151	48.4338	8.88 × 10^−10^
	STD	**0**	0	0	17.5878	3.3643	12.9391	4.26 × 10^−9^
	TIME	**0.1471**	0.1596	0.1512	0.9208	0.1983	0.1946	0.1685
F8	BEST	**0**	0	0	0.2068	3.39 × 10^−5^	1.7447	3.32 × 10^−8^
	WORST	**0**	0	0	0.9657	0.0305	6.2032	9.27 × 10^−7^
	MEAN	**0**	0	0	0.5836	0.0038	3.6787	2.92 × 10^−7^
	STD	**0**	0	0	0.2110	0.0082	1.3271	2.33 × 10^−7^
	TIME	**0.1697**	0.1883	0.1748	0.8457	0.2108	0.2174	0.1799
F9	BEST	**3.85 × 10^−4^**	9.66 × 10^−4^	0.0012	8.35 × 10^−4^	0.0132	1.8144	0.4098
	WORST	**0.1239**	0.1500	0.3743	0.9510	0.1933	10.3259	0.7856
	MEAN	**0.0425**	0.0591	0.0801	0.2765	0.0529	4.4220	0.5745
	STD	**0.0364**	0.0452	0.0838	0.2830	0.0417	1.9411	0.0823
	TIME	**0.3827**	0.3887	0.4158	1.1176	0.4226	0.5806	0.6227
F10	BEST	**1.21 × 10^−5^**	5.26 × 10^−4^	0.0015	0.1733	0.1694	29.1694	1.7590
	WORST	**1.5300**	1.6126	1.6774	4.8634	1.8458	7676.9234	2.9953
	MEAN	**0.3992**	0.4495	0.4977	1.3404	0.6365	944.6180	2.4436
	STD	**0.4592**	0.5537	0.4708	1.1938	0.4613	1705.6680	0.6278
	TIME	**0.3839**	0.4256	0.4051	1.1018	0.5169	0.4878	0.6235
F11	BEST	**3.09 × 10^−4^**	3.11 × 10^−4^	3.19 × 10^−4^	6.69 × 10^−4^	3.14 × 10^−4^	3.14 × 10^−4^	3.14 × 10^−4^
	WORST	**2.22 × 10^−3^**	2.29 × 10^−3^	3.07 × 10^−3^	0.0203	2.85 × 10^−3^	6.52 × 10^−3^	8.93 × 10^−3^
	MEAN	**8.46 × 10^−4^**	1.03 × 10^−3^	9.69 × 10^−4^	0.0190	6.02 × 10^−3^	2.07 × 10^−3^	4.77 × 10^−3^
	STD	**7.79 × 10^−4^**	2.57 × 10^−4^	8.56 × 10^−4^	0.0049	1.07 × 10^−3^	9.93 × 10^−4^	1.27 × 10^−3^
	TIME	**0.0787**	0.1009	0.1049	0.8153	0.1218	0.2401	0.2003
F12	BEST	**−10.5363**	−10.5336	−10.5363	−10.5363	−10.5361	−10.5363	−4.9747
	WORST	**−3.5611**	−2.6472	−1.7687	−2.8066	−3.1285	−2.8711	−1.9865
	MEAN	**−6.9625**	−5.5595	−5.6402	−4.3569	−6.4729	−6.2699	−3.4686
	STD	**1.0408**	2.7921	2.9755	3.1426	2.3719	2.3389	2.2510
	TIME	**0.1110**	0.1339	0.1355	1.0387	0.1788	0.2288	0.7654

**Table 4:** Wilcoxon signed rank sum test results.

Function	SPSOA-MSOA	SPSOA-BSOA	SPSOA-PSO	SPSOA-GWO	SPSOA-WSO	SPSOA-BOA
F1	NaN	NaN	1.10 × 10^−11^	1.10 × 10^−11^	1.10 × 10^−11^	1.10 × 10^−11^
F2	3.02 × 10^−11^	1.96 × 10^−5^	3.02 × 10^−11^	3.02 × 10^−11^	3.02 × 10^−11^	3.02 × 10^−11^
F3	3.02 × 10^−11^	3.02 × 10^−11^	3.02 × 10^−11^	3.02 × 10^−11^	3.02 × 10^−11^	3.02 × 10^−11^
F4	1.66 × 10^−4^	2.47 × 10^−4^	3.02 × 10^−11^	2.27 × 10^−5^	3.02 × 10^−11^	3.02 × 10^−11^
F5	1.85 × 10^−4^	1.17 × 10^−4^	4.45 × 10^−4^	3.33 × 10^−4^	3.02 × 10^−11^	3.02 × 10^−11^
F6	2.92 × 10^−4^	3.62 × 10^−4^	3.01 × 10^−11^	1.10 × 10^−8^	3.01 × 10^−11^	3.01 × 10^−11^
F7	NaN	NaN	1.21 × 10^−12^	4.26 × 10^−12^	1.21 × 10^−12^	1.21 × 10^−12^
F8	NaN	NaN	1.21 × 10^−12^	5.58 × 10^−4^	1.21 × 10^−12^	1.21 × 10^−12^
F9	6.54 × 10^−4^	1.17 × 10^−5^	3.32 × 10^−6^	4.52 × 10^−4^	3.02 × 10^−11^	3.02 × 10^−11^
F10	8.31 × 10^−4^	2.29 × 10^−4^	6.73 × 10^−6^	6.35 × 10^−5^	3.02 × 10^−11^	3.02 × 10^−11^
F11	3.32 × 10^−11^	3.01 × 10^−11^	3.33 × 10^−11^	3.68 × 10^−11^	3.01 × 10^−11^	3.01 × 10^−11^
F12	1.07 × 10^−11^	1.07 × 10^−11^	1.07 × 10^−11^	1.07 × 10^−11^	1.07 × 10^−11^	1.07 × 10^−11^
+/=/−	9/3/0	9/3/0	12/0/0	12/0/0	12/0/0	12/0/0

**Table 5:** Data of image signal separation performance evaluation index.

Algorithm	SOA	MSOA	BSOA	SPSOA
similarity coefficient	0.8574	0.9052	0.9240	0.9784
	0.8909	0.8793	0.9065	0.9638
	0.8445	0.8961	0.9457	0.9857
	0.8283	0.9178	0.9247	0.9863
PI	0.2786	0.2031	0.1549	0.1127
SSIM	0.8233	0.8764	0.9147	0.9592
